# Meningomyelocele Simulation Model: Pre-surgical Management–Technical Report

**DOI:** 10.7759/cureus.2231

**Published:** 2018-02-26

**Authors:** Orna Rosen, Robert M Angert

**Affiliations:** 1 Neonatology, Pediatrics, Children's Hospital at Montefiore, Montefiore Medical Center

**Keywords:** myelomeningocele, delivery room, pre-surgical management, resuscitation, newborn, neural tube defects

## Abstract

This technical report describes the creation of a myelomeningocele model of a newborn baby. This is a simple, low-cost, and easy-to-assemble model that allows the medical team to practice the delivery room management of a newborn with myelomeningocele. The report includes scenarios and a suggested checklist with which the model can be employed.

## Introduction

Myelomeningocele is a congenital anomaly with an incidence of 2.8/1000 births [1]. Appropriate delivery room care for these newborns is critical for an optimal outcome. The exposed membranes on the back can easily rupture even with minor trauma, exposing neural tissue and leading to meningitis. The resuscitation of a baby with myelomeningocele is challenging and, therefore, appropriate preparations, special equipment, and adequate experience are needed. Having the opportunity to practice the procedure in a simulated environment allows trainees to gain valuable experience prior to being in a live situation. Creating a model that is realistic enough to immerse the trainee in the scenario allows individuals and teams an opportunity to practice, gaining valuable skills and knowledge prior to handling a patient with this condition in the delivery room.

## Technical report

Preparing the materials

**Figure 1:**
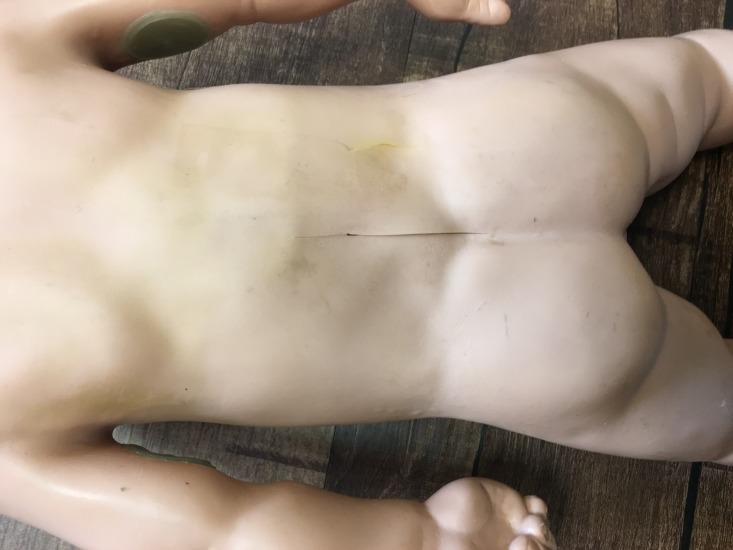
Model with 5-cm incision on the back

The simulation doll used in this model was an older rubber model (Simulaids, Inc., Saugerties, NY, US (1978)). Any doll with a rubber or plastic body that is the approximate size of a newborn would suffice. A longitudinal slit, 5-cm long, was created along the location of the spine in the lumbosacral area using a surgical scalpel, such as a #11 blade (Figure 1). 

**Figure 2:**
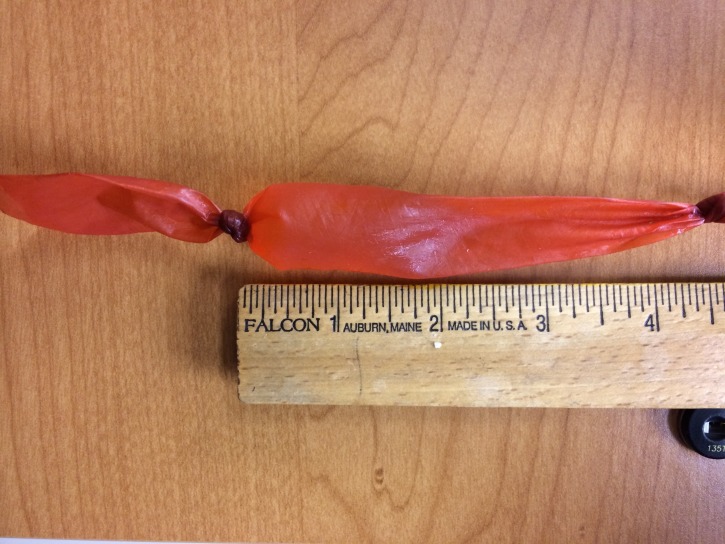
Sausage casing with water, knotted at both ends

A 21-millimeter diameter collagen sausage casing (Snack Stick Collagen Casing, Smokehouse Chef, Amazon.com) was used to create the simulated myelomeningocele. We divided the casing into three 20-cm long pieces. It was soaked in water to make it soft and flexible, a knot was placed at one end, the casing was filled with water to a height of 5 cm and a second knot was tied at 10-11 cm (approximately 4 inches) from the first knot (Figure 2).

Assembly of the simulator

**Figure 3:**
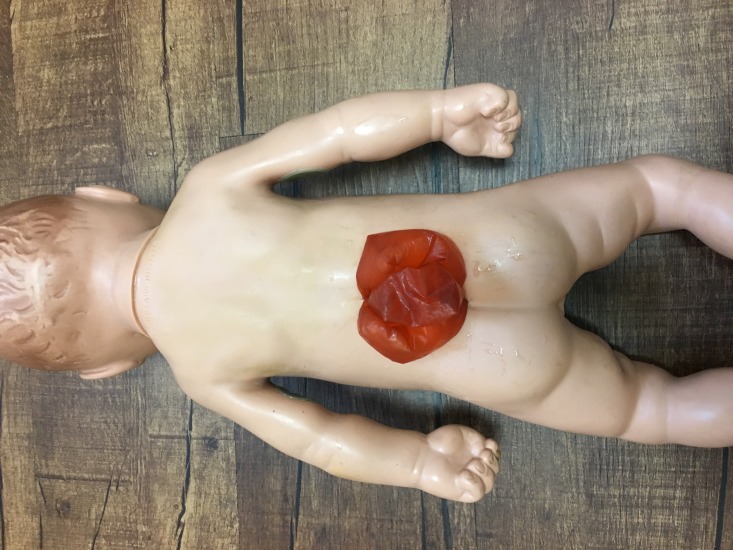
Large meningomyelocele with three loops

To create the myelomeningocele, we used the above-mentioned sausage casing. The most realistic appearance was achieved with three loops of the sausage casing filled with water and double-knotted as described. We inserted the three loops filled with water into the slot in the doll’s back, creating the simulated membrane-covered lesion (Figure 3).

**Figure 4:**
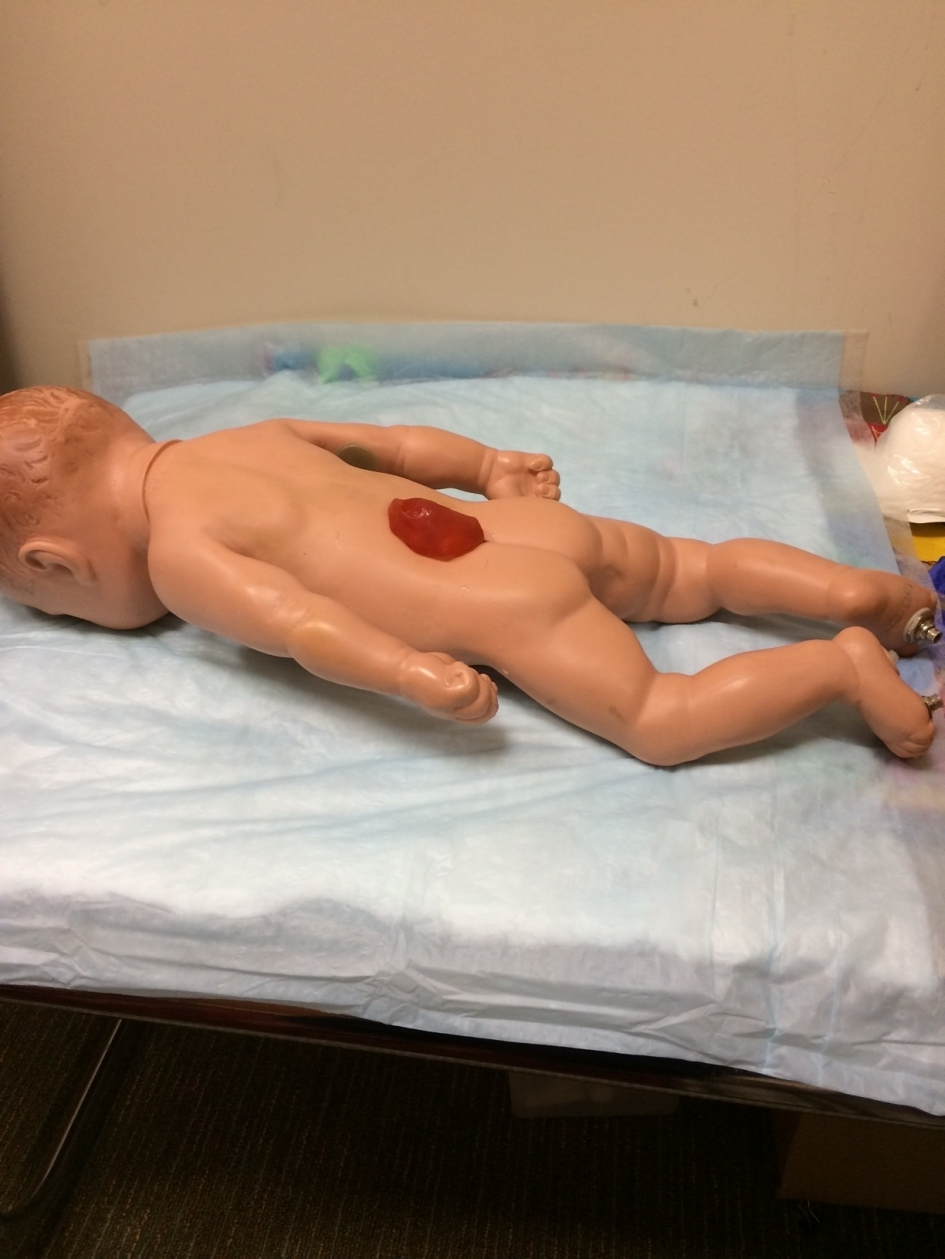
Small meningomyelocele with one loop

 A smaller or single segment of water-filled collagen sausage casing can be used to simulate different sized or shaped lesions (Figure 4). 

Learning objectives

The simulation instructor will assemble and demonstrate our inexpensive model of a newborn with myelomeningocele.

Trainees and providers will master the delivery room handling of newborns with myelomeningocele in the following way:

1. Maintain a sterile, latex-free environment.

2. Place the baby in a side-lying position to avoid pressure on the back lesion.

3. Wrap the lesion in a sterile, saline-soaked gauze with an occlusive plastic wrap.

4. Intubate the baby without putting pressure on the lesion.

Additional Supplies for the Scenario

Sterile latex-free gloves

Sterile gauze

Warm sterile saline

Saran wrap

Sponge donut

Fluidized positioner (Z-Flo, Sundance Enterprises, Medline Industries, Illinois, United States)

Endotracheal tube (ETT), stylet, laryngoscope, suction catheter/suction bulb, carbon dioxide detector, stethoscope, and adhesive tape

Sample Critical Action Checklist for the Resuscitation of a Baby with Myelomeningocele

❏ Gather and prepare supplies, maintaining a sterile, latex-free environment.

❏ Delegate tasks to providers: Hand the baby over and receive the newborn. Position the newborn, place the pulse oximeter probe, wrap and protect the lesion, manage the airway, and obtain IV access, if needed.

❏ Initial steps: Place baby on its abdomen, dry the upper body of the baby, and carefully dry the legs, avoiding pressure on the lesion. Perform oral and nasal suctioning, if needed.

**Figure 5:**
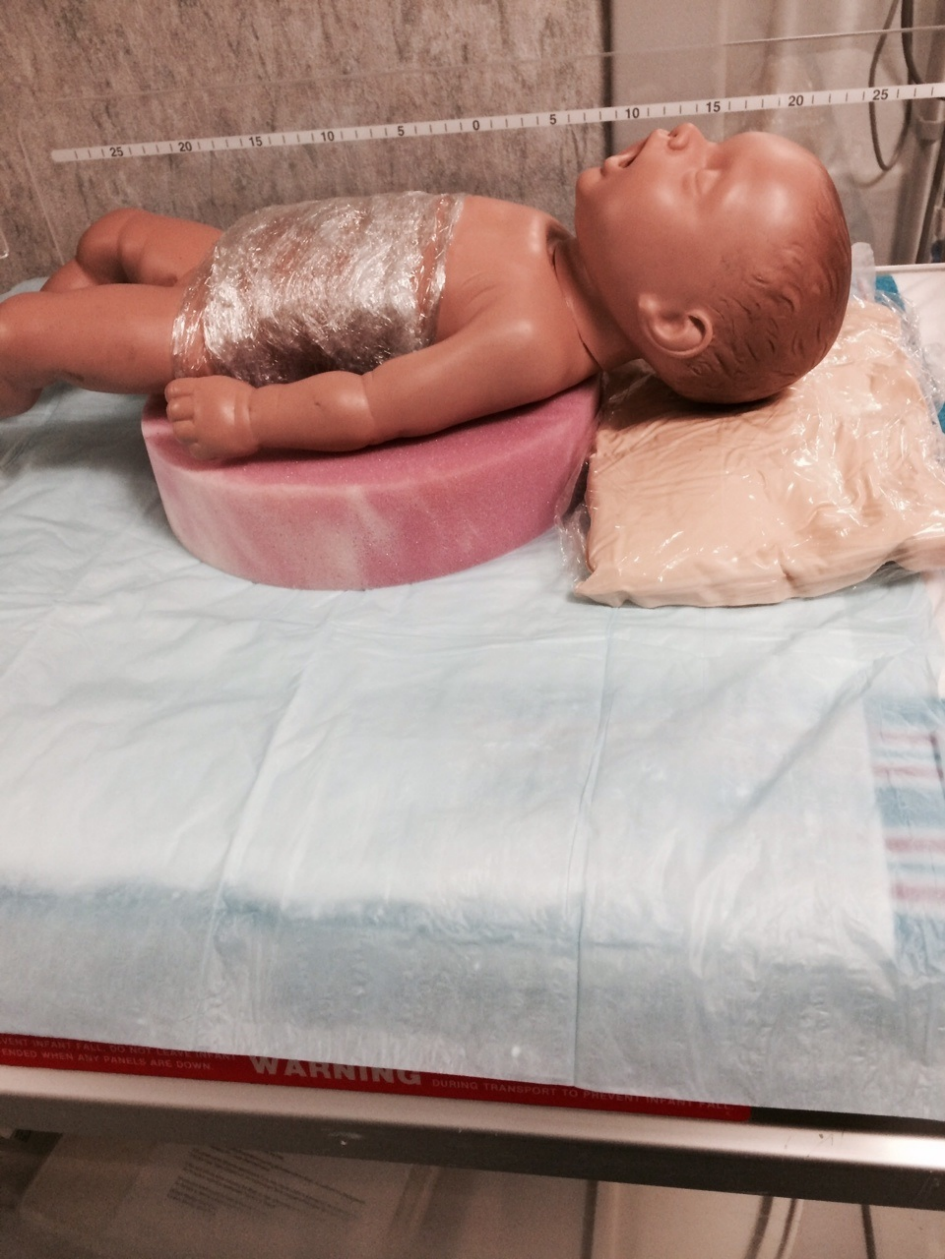
Simulator positioned on the donut with the fluidic positioner

❏ Wrap the lesion with sterile gauze soaked in warm saline (Figure 5).

❏ Cover with dry sterile gauze.

❏ Cover the lesion and abdomen with layers of occlusive plastic wrap.

**Figure 6:**
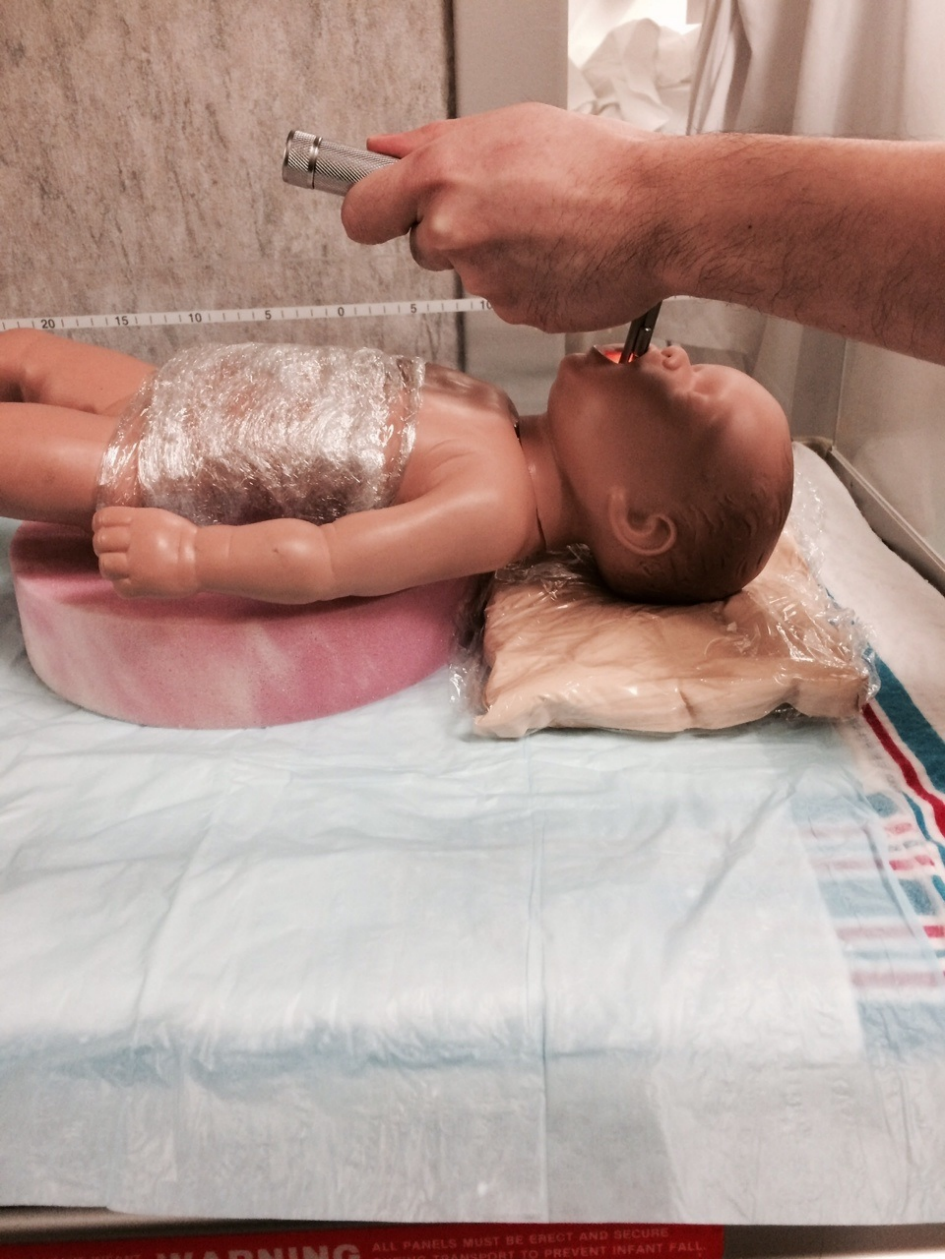
Simulator with meningomyelocele protected, being intubated

❏ If positive pressure ventilation and/or intubation is needed, put the baby in the prone position with the simulated lesion placed inside the donut hole (Figure 6).

**Figure 7:**
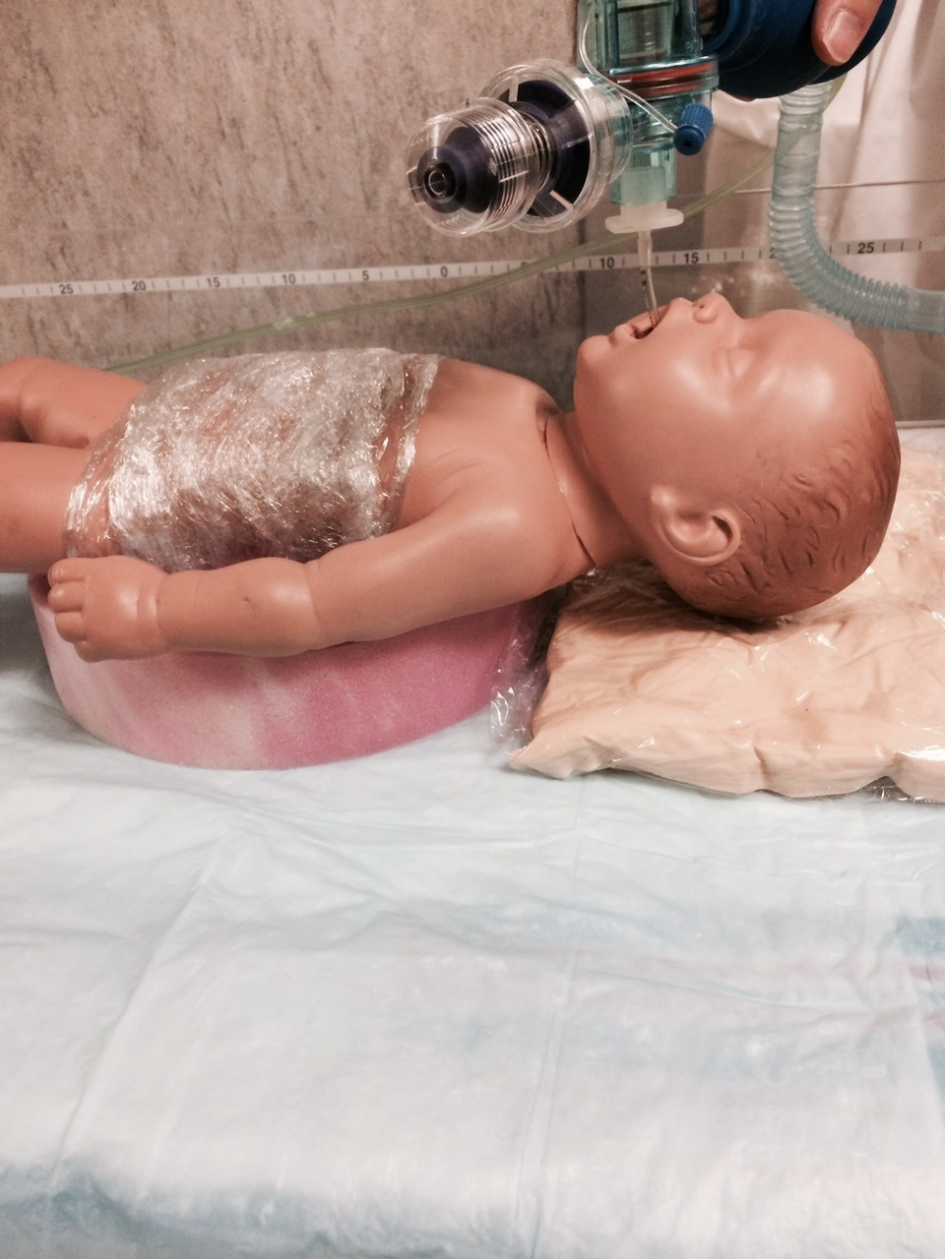
Simulator with meningomyelocele protected, ETT in place ETT: endotracheal tube

❏ Support the baby’s head with a fluidized positioner or with the sponge donut center (Figure 7).

❏ Intubate and secure ETT.

Clinical scenario: myelomeningocele

Background: A pregnant woman at 41 weeks' gestation is admitted to the hospital with the onset of regular contractions and cervical changes. Her prenatal labs are negative, including Group B strep testing. Findings from a prenatal sonogram indicate myelomeningocele. The anomaly was discovered at a 26-week ultrasound, as the mother was a late registrant with prenatal care. Fetal monitoring reveals a category II tracing, and the decision is made by the obstetrician to perform a cesarean section. When the membranes are ruptured, there is meconium-stained amniotic fluid. The baby emerges cyanotic with good tone and a spontaneous cry.

Course: Follow the critical action checklist given above.

Option A: Routine steps are given and the baby responds with spontaneous respirations and the color improves. The heart rate is in the 120s.

Option B: The baby develops respiratory distress with severe retractions and cyanosis. There is decreased air movement and the heart rate decreases to 50. The baby is changed to the supine position, while the circular foam ‘donut’ is placed under the baby to protect the back lesion by positioning it inside the donut hole. A fluidized positioner is placed under the model's head to support it during the procedure. The airway should be cleared and the baby should be given positive pressure ventilation. There is no response and attempts are made to improve the positive pressure ventilation (mask reposition suction open mouth pressure increased alternative airway – MR SOPA). There is no improvement, and the baby requires endotracheal intubation. After successful endotracheal intubation, the heart rate, color, and tone improve.

## Discussion

This model had been used three times per year for five years to train fellows, nurse practitioners, physician assistants, and attending physicians. The providers report that "It was very realistic. I loved the hands-on way we learned and how we were so well-prepared for the real delivery room experience." "It was great to go through different scenarios. We were able to use the same materials that were used in actual deliveries." "This practice was very helpful for real life!"

We give two suggested scenarios:

Option A requires minimal intervention, but it allows the trainees to focus on maintaining sterility, maintaining a latex-free environment and preventing injury to the lesion. This is useful for the novice provider who needs basic practice with handling a baby with this condition. It also can be employed to focus on teamwork and communication by allowing an analysis of team behaviors. The scenario can be employed as just-in-time training for teams when a meningomyelocele delivery is anticipated and practice is desired prior to the actual delivery [2].

Option B adds respiratory depression to the scenario and allows the provider to practice intubating a baby with meningomyelocele in a simulated environment. Hydrocephalus, a frequent complicating condition in meningomyelocele, can be discussed in the debriefing. The suggested positional management, including the foam donut and Z-Flo (EdiZONE, LLC, Utah, USA) pillows, can be arranged to accommodate different head shapes and sizes. It is also possible to wrap the head of the model with gauze to simulate a larger head circumference. Intubating a baby with meningomyelocele is a very challenging maneuver to perform while protecting the spinal lesion. Institutions can adapt the model to their own practice by modifying the positioning aids or trialing new devices and materials. Other variations of the scenario can be used to meet different learning objectives, such as learning to manage shock in a patient with meningomyelocele, requiring central line placement, fluid boluses, and epinephrine administration.

Patients with open spinal lesions are at high risk of developing latex sensitization [3]. Avoiding latex exposure is the primary method for preventing latex sensitivity. This simulation model and suggested scenario allow learners to practice the setup and resuscitation, paying careful attention to avoid the use of latex-containing materials. It is possible to employ a confederate to bring a latex-containing product and test the team’s situational awareness.

 Since the delivery of a neonate with meningomyelocele is relatively rare even in the busiest programs, simulation-based training affords the advantage of allowing many individuals to practice their skills and learn to work in teams to provide optimal care. Obstetricians can participate in scenarios so that the delivery and hand-off can be practiced in a safe, simulated environment with the opportunity to debrief and reflect. Neurosurgeons, who rarely participate in the resuscitation due to logistical constraints, can have input in standardizing and improving care for the spinal lesion during the resuscitation, allowing the patient to arrive in the best possible condition prior to surgical intervention [4].

## Conclusions

This model and the accompanying suggested scenarios allow programs to create an inexpensive, simple-to-assemble simulator that can be used to teach individual skills as well as to test the performance of teams who are called on to provide newborn care for neonates with complex spinal lesions. The ability to include a wide range of provider training levels and a multidisciplinary group will lead to better overall performance and patient outcomes.
